# A quantitative non-invasive assessment of femoroacetabular impingement with CT-based dynamic simulation - cadaveric validation study

**DOI:** 10.1186/s12891-015-0504-7

**Published:** 2015-03-11

**Authors:** Maarten A Röling, Monique I Visser, Edwin HG Oei, Peter Pilot, Gert-Jan Kleinrensink, Rolf M Bloem

**Affiliations:** Department of Orthopedic surgery, Reinier de Graafweg 3-11, 2526 AD Delft, Netherlands; Department of Radiology Erasmus Medical Center, Rotterdam, Netherlands; Department of Neuroscience, Erasmus Medical Center, Rotterdam, Netherlands

**Keywords:** Femoroacetabular impingement, Diagnostics, CT, Dynamic

## Abstract

**Background:**

Femoroacetabular impingement (FAI) is caused by an anatomic deviation of the acetabular rim or proximal femur, which causes chronic groin pain. Radiological identification of FAI can be challenging. Advances in imaging techniques with the use of computed tomography (CT) scan enable 3D simulation of FAI. We made an experimental cadaveric validation study to validate the 3D simulation imaging software.

**Methods:**

The range of motion (ROM) of five cadaveric hips was measured using an electromagnetic tracking system (EMTS). Specific marked spots in the femur and pelvis were created as reproducible EMTS registration points. Reproducible motions were measured. Hips were subsequently imaged using high-resolution CT after introduction of artificial cam deformities. A proprietary software tool was used, Articulis (Clinical Graphics) to simulate the ROM during the presence and absence of the induced cam deformities.

**Results:**

According to the EMTS, 13 of the 30 measured ROM end-points were restricted by > 5° due to the induced cam deformities. Using Articulis, with the same 5° threshold, we correctly detected 12 of these 13 end point limitations and detected no false positives. The median error of the measured limitations was 1.9° (interquartile range 1.1° - 4.4°). The maximum absolute error was 5.4°.

**Conclusions:**

The use of this dynamic simulation software to determine the presence of motion limiting deformities of the femoroacetabular is validated. The simulation software is able to non-invasively detect a reduction in achievable ROM, caused by a cam type deformity.

## Background

Femoroacetabular impingement (FAI) is an accepted etiology of premature osteoarthritis of the non-dysplastic hip [1]. It has predominance for males, with a prevalence of 17% in men and 4% in women [2,3]. FAI caused by a cam or pincer deformity can be treated by open dislocation and osteotomy, mini-open procedure or by an arthroscopic resection of the bony deformity. All methods are effective at reducing pain, improving function and are relatively save. The arthroscopic method has a lower complication rate and functional results of this procedure have been described as good [4-7]. Also, a high return to pre-injury levels of sports performance in athletes has been described [8]. However, not all patients recover as to be expected, and revision of the arthroscopy may be needed in these cases. Persistent bony impingement due to residual or untreated bone deformity of the hip and underlying osteoarthritis have been described as the most frequent causes of revision arthroscopy, up to 95% [9-11] It is, therefore, of paramount importance to diagnose the exact position of the deformity causing the impingement. Plain radiography, computed tomography (CT) and magnetic resonance imaging (MRI) are commonly used in the common diagnostic work-up of FAI. Despite this variety of radiological modalities, it remains a challenge to comprehensively evaluate the FAI associated deformities and, thus, to create a complete resection of the FAI. Several authors have pointed out the inefficacy of the current morphological parameters on plain radiographs [12-15]. We still lack methods to determine whether a deformity impinges during movements of a patient. Evaluation by clinicians remains an important part of diagnosing FAI [16]. Dynamic evaluation can be helpful in determining whether impingement occurs.

Recent advances in 3D imaging enable simulation of range of motion (ROM) of joints in patients [17]. By converting image data to virtual 3D models of the femur and the pelvis, it is possible so simulate the dynamic function of a hip joint. Used in conjunction with the clinical examination of the hip joint, these motion simulations may confirm whether groin pain is attributable to morphological characteristics of the joint [18].

The aim of this study was to validate a CT-based motion simulation software method that has already been validated for other joints [16,19], in the context of FAI, and to evaluate the method’s applicability for the diagnostic work-up of FAI in a prospective cohort follow-up study of FAI patients. Although this software has perfect repeatability, it is no golden standard for measurement of range of motion. For this purpose, we determined its accuracy in a range of motion assessment study of five human cadavers. We hypothesized that the software is a reliable measurement tool to detect a reduction in achievable ROM caused by a cam type deformity.

## Methods

Five human cadaveric hip joints from three individuals who had donated their bodies to science (two female, one male) were available from the Department of Anatomy (institution blinded). All anatomic specimens were prepared with Anubifix™ (city blinded, the Netherlands) for optimal preservation [20] and selected for absence of obesity, lack of a total hip arthroplasty and an optimal flexibility of the hip joint of at least 90° of flexion. Gender or age was no selection criteria.

**Figure 1:**
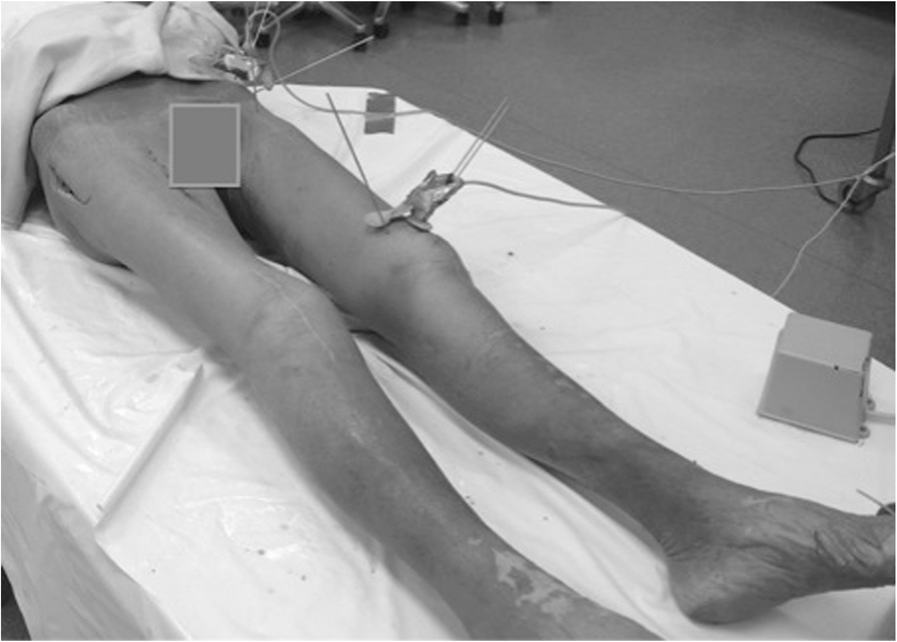
**The positioning of the cadaver in supine position with the Kirschner-wires in position.** Next to the left foot is the main device of the EMTS.

In order to expose the hip joint and to maintain stability and flexibility all the cadaveric hip joints were prepared with the anterolateral approach according to Hueter [21] (Figure 1).

**Figure 2:**
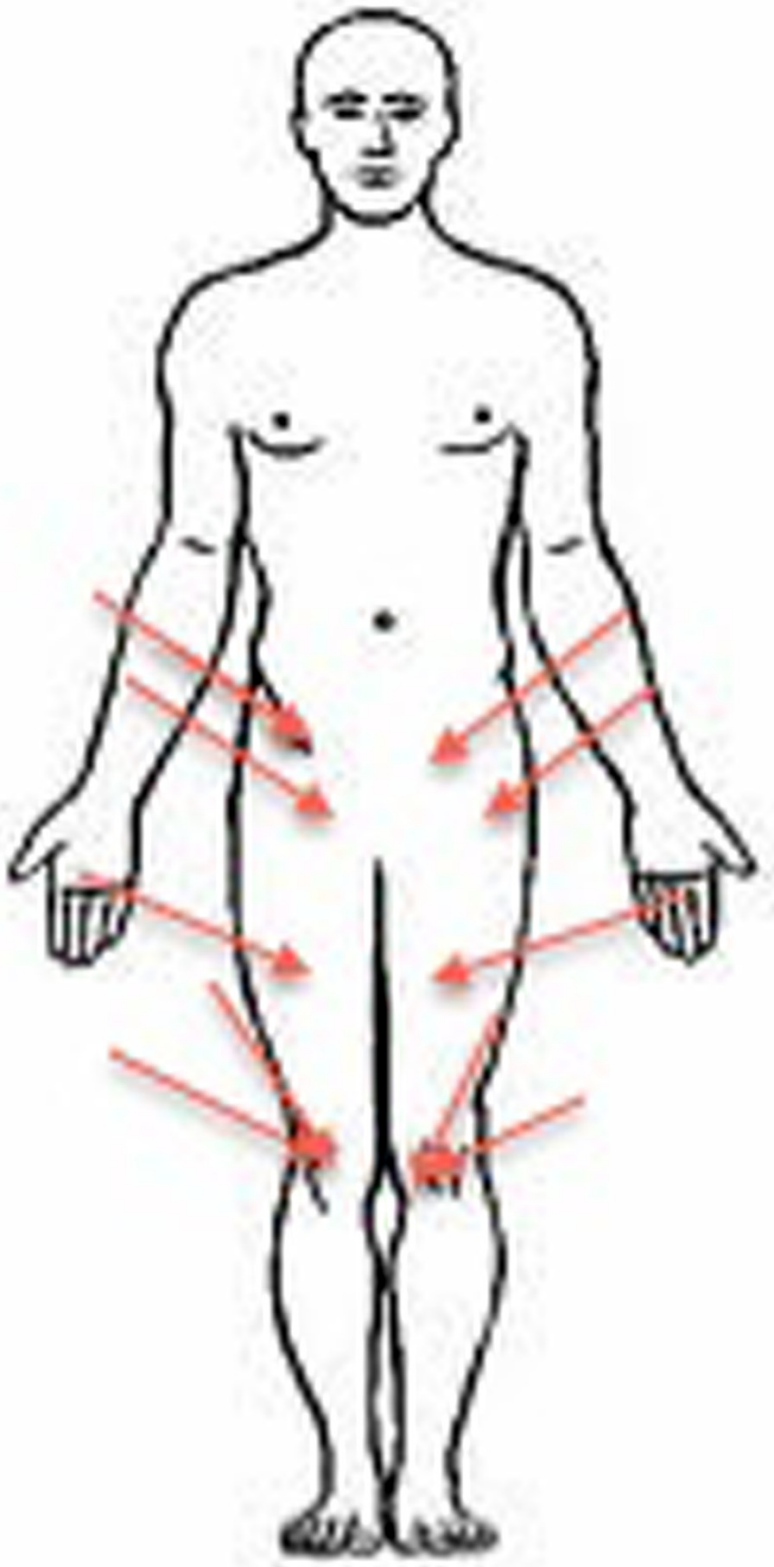
**The position of the K-wires in the specimen.**

Measurements of the range of motion of the hips were acquired using the electromagnetic tracking system [22] (EMTS), (Flock of Birds, Ascension Technology, United States). This system uses a magnetic field to determine the position and orientation of the sensors to its transmitter. The system requires reproducible registration points on the hip and the femur, according to the ISB recommendations coordinate system. Kirchner-wires were attached into every specimen on marked locations: two K-wires, three centimeters apart, were positioned into the superior anterior iliac spine as registration points for the pelvis. One K-wire was attached into the greater trochanter and one in each epicondyles of the knee as the registration points of the femur (Figure 2). The sensors were attached to the K-wires, as close to the skin as possible. A final sensor was attached to a pointer that registered the other sensors. As per the guidelines of Milne [23], optimal range between the transmitter and the sensors should be between 22.5 and 64.0 cm. All specimens were prepared and measured on a plastic table and all metal objects within a range of one meter were removed to prevent interference with the magnetic signal.

We registered the maximum flexion, abduction, internal rotation at 0°, 30°, 60° and 90° of flexion. Any positional and rotational changes of respectively 0.25 mm and 0.1° were determined. We measured all end points twice and all differences were < 2%.

**Figure 3:**
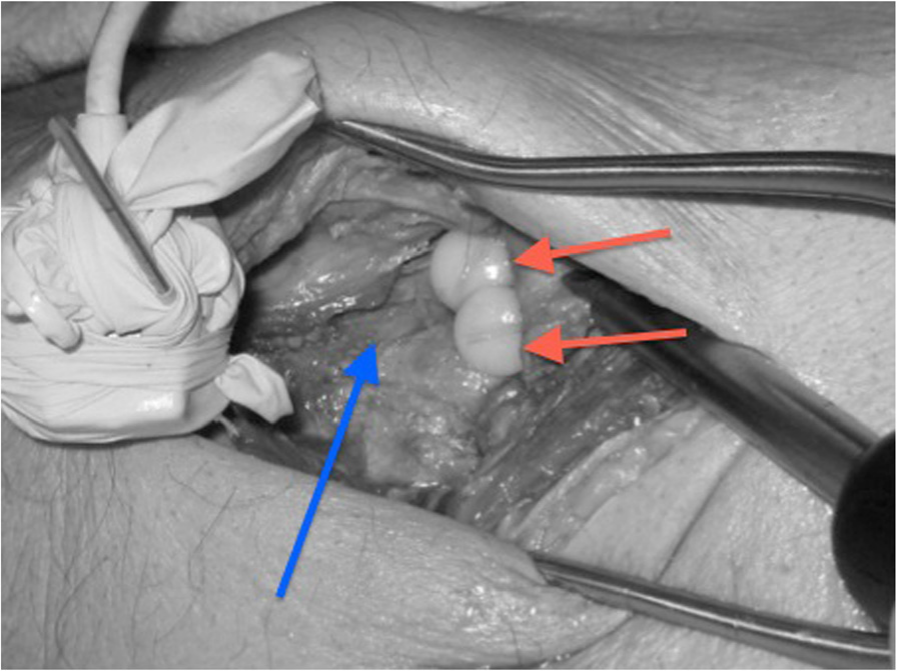
**Artificial cam deformity created by nylon screws (red arrow pointing at the screws) inserted at the anterio-superior position of the head-neck junction, after preparation with the Hueter approach.** The blue arrow points at the collum of the femur.

An artificial cam deformity was created using nylon screws with a diameter of 1 cm and a thickness of 3.5 mm (Figure 3). Nylon was used because it is known not to interfere with the EMTS while it provides sufficient contrast on the CT images. The density of nylon is less than human bone: 1.15 g/cm^3^ vs. 1.9 g/cm^3^ respectively, and can be distinguished from bone and surrounding soft tissues. Two screws were inserted on the anterio-superior position of the femoral head, between the 11 and 2 ‘o clock-position in full extension and neutral position of the hip [24,25]. After insertion of the screws, the exact same measurements were taken as before. As the simulation software does not take into account the soft tissues of the joint and thus over-estimated the range of motion of each hip joint by default, we chose not to assess the absolute range of motion but the relative change in range of motion as a result of an introduced cam deformity.

The specimens were subsequently imaged by means of non-contrast CT scan. CT scan was performed in the Department of Radiology, (institution blinded), using a second generation dual source multidetector spiral CT scanner (SOMATOM Definition Flash, Siemens Healthcare AG, Erlangen, Germany) with a tube voltage of 80 kV and an effective mAs-value of 3,140. Scan time per CT scan was approximately 30 seconds. All specimens were scanned in the standard anatomic axial plane orientation and were reconstructed with an effective slice thickness of 1.0 mm and a sharp reconstruction kernel (B75s). Multi-planar reconstruction was performed (image pixel size 0.265 mm).

We used the software package Articulis (institution blinded, city blinded, the Netherlands) to simulate the ROM of the hip joints. The software used the introduced k-wires as registration points, so that the measurements were exactly reproducible between the CT-model and the specimens. The software then automatically converts the CT scans to 3-Dimensional models of the femur and the pelvises. For each hip joint, two different versions of the femur were created: one with and one without the artificially induced cam deformities. The software identifies the impinging area by 0.1 mm and calculates the amount of bone necessary to resect and dissolve the impingement.

**Figure 4:**
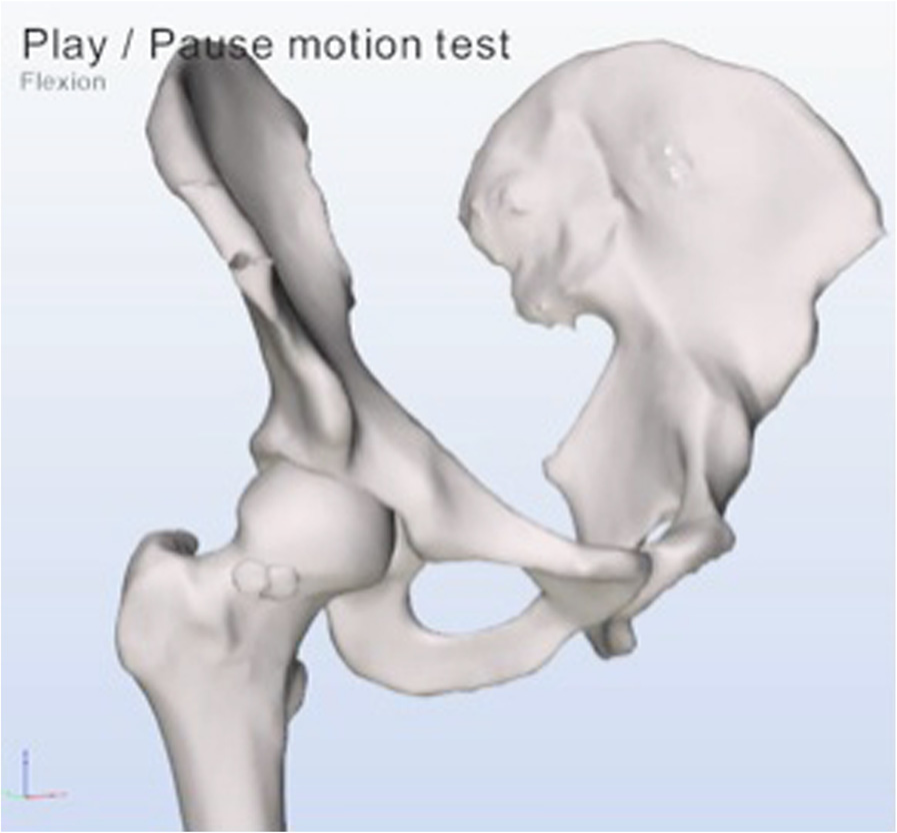
**A simulation of the artificial cam deformity by the software with the hip in extension: the cam deformity is clearly visible at the anterio-superior position at the head-neck edge.**

**Figure 5:**
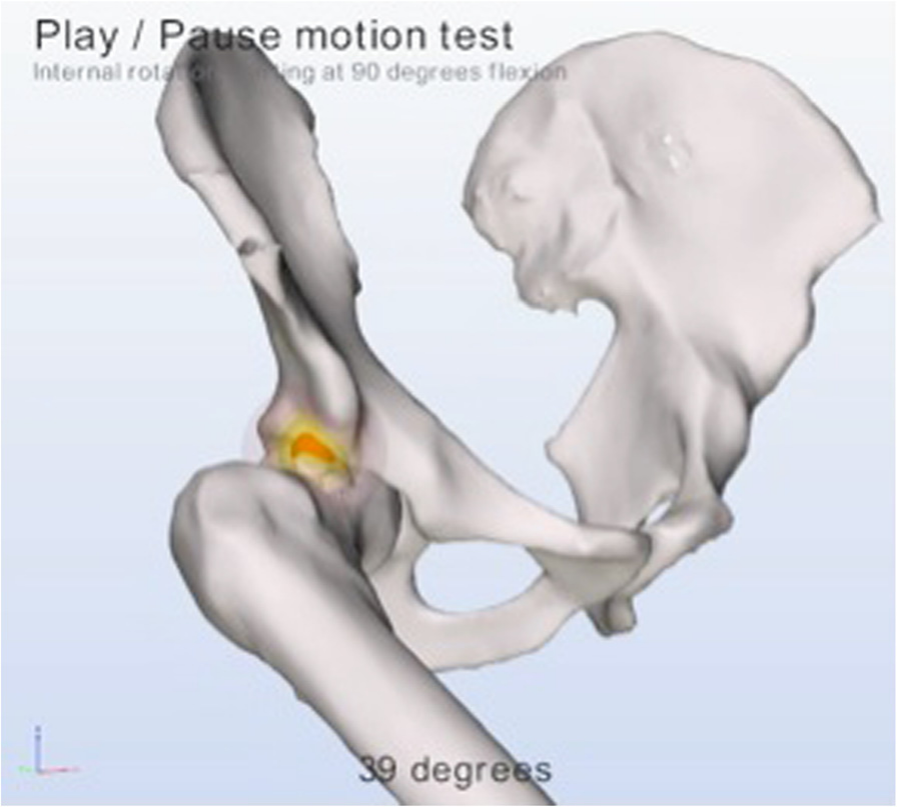
**A simulation of internal rotation made by the software, which detects an impingement of the cam deformity with the acetabulum and therefor limiting the internal rotation.**

Articulis uses the coordinate systems as described in the Recommendations of the International Society of Biomechanics [26] and the equidistant method described by Puls et al. in [27]. Flexion refers to elevation parallel to the sagittal plane along the Z-axis of the pelvis. Abduction refers to elevation in the coronal plane along the X-axis of the pelvis and internal rotations refer to axial rotation along the femur shaft of Y-axis of the femur. The software systematically simulates different motions, for example flexion, abduction, internal rotations with 90° flexion. While reorienting the femur model the software checks for collisions of the bone models. Up to 3 mm of translation of the femoral head is allowed, applied when reorienting the femur leads to collisions. When more than 3 mm of translation is required to reach a collision free state, simulation is halted and the angle at which impingement occurred is registered. Figures 4 and 5 are simulations as provided by the software of a cam deformity causing impingement during simulated internal rotation.

No ethics approval was obliged for this cadaveric validation study.

### Statistical analysis

We compared the motion limitations observed with the EMTS to the motion limitations determined by the Articulis ROM simulation software. The median deviation, interquartile range and the maximum absolute deviation of the differences were calculated. For these calculations the measurements of the five cadaveric hip joints were combined. Statistical analysis was performed using IBM SPSS Statistic 20 (SPSS, Chicago, Illinois).

## Results

According to the EMTS, 13 of the 30 measured ROM end points were restricted by more than 5° due to the induced cam deformities. Using Articulis and with the same 5° threshold, we correctly detected 12 of these 13 end point limitations and detected no false positives. The median error of the simulated ROM limitations compared to the EMTS measured limitations was 1.9° (interquartile range 1.1° - 4.4°). The maximum absolute error was 5.4°. Table 1 is an example of our measurements of a cadaveric hip joint. Table 2 summarized all measurements.

**Table 1:** **Example of comparison measurements software vs. EMTS of hip joint 5**

	**Simulations**			**EMTS**			
	**No Cam**	**Cam**	**Difference**	**No Cam**	**Cam**	**Difference**	**Difference of the difference**
**Max flexion**	105	93	12	110	93	16	−4
**Max abduction**	29	31	0	35	34	1	−1
**Max internal rotation**	60	60	0	38	37	1	−1
**Max internal rotation at 30°**	34	34	0	32	34	−2	2
**Max internal rotation at 60°**	27	26	0	37	31	6	−5
**Max internal rotation at 90°**	20	1	19	32	18	14	5

**Table 2:** **Difference in degrees between the true limitation (measured with EMTS) and the limitation detected by the simulation software**

**Hip joint**	**1**	**2**	**3**	**4**	**5**	
**ci**	**R**	**R**	**L**	**R**	**L**	**Median**
**Max flexion**	−2	−1	−2	−2	−4	−1.7
**Max abduction**	−1	0	1	−1	−1	−0.5
**Max internal rotation**	0	3	−7	−2	−1	−0.8
**Max internal rotation at 30°**	0	−1	2	−1	1	0.1
**Max internal rotation at 60°**	−1	−1	5	−2	−5	−1.3
**Max internal rotation at 90°**	02	1	0	−3	5	0.0

## Discussion

In this cadaveric study we evaluated the presence of motion limiting deformities of the femoroacetabular joint by non-invasive modeling software, using 3D radiological imaging. We correctly detected 12 of the 13 end point limitations compared to the EMTS as the gold standard for measurements of ROM. The one hip that we could not correctly detect was mainly limited by soft tissue problems of the hip, which totally limited the internal rotation in neutral position. With a median error of only 1.9°, we can consider the software a highly reliable measuring tool.

Based on our study, we consider this CT-based motion simulation software as validated in the context of measuring the ROM of a hip joint that are limited by FAI deformities.

We chose a 5° threshold to evaluate if the software could detect such a limitation. A 5° threshold is fare above the measurement error (1,9°) of the software. We don’t state that this 5° limitation also is a significant limitation of the motion of the hip. This amount is only set to evaluate the accurateness of the software in detecting changes in the range of motion caused by an impinging deformity.

The use of cadaveric hip joints has its limitations and disadvantages. All specimens were prepared with Anubifix^TM^ for optimal preservation [20]. Despite being optimally preserved, joints flexibility or ROM is not identical as in a living human. The use of specimens was inevitable to be able to prepare a standardized artificial cam deformity in the hip joint. By creating the cam deformity, we had exact information about the size and position of the deformity. This knowledge provided us with an accurate ground truth to compare our simulations against. We consider our method of comparing our standardized measurements of the deformities to their exact parameters as very accurate. The expected limited ROM of the cadavers did not influence our measurements, as the movements of the hips during our measurements were not limited by stiffness of the soft tissues of the cadavers.

Our purpose was to determine whether the simulations of the software could accurately determine ROM as encountered in physical examinations. We used the EMTS “Flock of Birds” system as a gold standard for the measurement of movement and angulation. This system has been calibrated and validated for many applications in motion measurements. Comparing the angles of the ROM with and without an impinging cam-type deformity of both methods demonstrates that the software correctly assesses ROM.

To compare the measurements of the software to the movements of the hip in real life, during sports-activities for example, was not the goal of this study. Our goal was to determine whether the measurement were reliable and valid. The software is able to determine every kind of range of motion possible in the joint. We didn't measure complicated combined angles, which are needed in real life sports like field hockey or soccer, because our specimen weren’t able to provide such range of motion. This is a limitation of our study and due to the specimen we used. If we had determined what kind of combined movements the hip joint makes during sports, than the software should be able to reproduce these combined movements. However, if the hip joint is limited at the ranges we measured, then it would probably also be limited during sports which requires a larger free range of motion.

Visualization of the cam deformity causing a FAI is challenging. Plain radiography with measurement of alpha angles as well as high resolution and multiplanar CT are widely used. Because of the dynamic aspect of FAI it is nearly impossible to detect the exact impinging location on a two dimensional image. Although Barton et al. [28] and Nepple et al. [29] state that the use of the alpha angle in the evaluation of cam-type FAI is validated, the use of CT scans adds an essential third dimension. But even in this gold standard for diagnostics, the dynamic aspect remains neglected. Several authors support this flaw of the alpha angle measurement [12-15]. Also, a recent study by de Bruin et al. [30] describes a very high prevalence of radiographic signs of FAI at all ages in an asymptomatic population (up to 86.59%). This emphasizes the importance of a simulated analysis based on these radiographic images or direct kinematics analysis.

CT scans have the disadvantage of ionizing radiation. The appropriateness of the use of CT scans should therefore always be evaluated. Accurate diagnosing could, however, limit the amount of unsuccessful operations and revision arthroscopies of the hip joint for FAI. We believe that the use of non-contrast based CT scans for diagnosing FAI is acceptable because there is currently no true alternative. Low dose reduction techniques and, as described by Gervaise in 2013 [31] and low dose protocols as described by Becce et al. in 2013 [32], might be solutions for these radiation problems. Further research in this area must point out if these alterations compromise the quality of possible dynamic analyses. Magnetic resonance imaging (MRI) has the potential to be a good alternative, as it does not involve radiation. MRI is, however, more challenging for three-dimensional simulation of the joints due to a lower spatial resolution and less accurate delineation of bone compared to CT with most MRI pulse sequences. Besides image acquisition with MRI requires more time than for CT.

The validation of this software opens up the possibility to use dynamic motion simulation based on CT scans in the diagnostic pathway or FAI. We hypothesize that creating a dynamic model will result in better functional outcomes in patients with FAI compared to those in previous studies. Described functional outcome results of FAI treated by hip arthroscopy are good [4,7]. The rate of unsuccessful surgeries and revision-surgeries could be diminished due to better visualization of the deformity causing the impingement. This hypothesis is currently under investigation by adding the CT movement analysis to our diagnostic work-up for FAI in the off setting of our prospective cohort, which is currently under analysis.

## Conclusions

This cadaveric study evaluated the use of software to determine the presence of motion limiting deformities of the femoroacetabular joint using radiological imaging with CT. To our knowledge, this is the first study to validate a non-invasive dynamic simulation on pre- and post-operative scenarios representing cam type deformities. The simulation software is able to non-invasively detect a reduction in achievable ROM, caused by a cam type deformity. This technique shows promise as a clinically diagnostic tool for FAI diagnostics and for preoperative planning.

## Abbreviations

FAI: Femoroacetabular impingement
CT: Computed tomography
ROM: R of motion
EMTS: Electromagnetic tracking system
MRI: Magnetic resonance imaging
